# Organ Support Requirements as Markers of Disease Severity and Mortality in Hospitalized Patients

**DOI:** 10.3390/jcm15051766

**Published:** 2026-02-26

**Authors:** Carmen Pantis, Mihaela Simona Popoviciu, Timea Claudia Ghitea, Manuela Alina Pop, Roxana Daniela Brata

**Affiliations:** 1 Department of Medical Disciplines, Faculty of Medicine and Pharmacy, University of Oradea, 1 Decembrie, 410028 Oradea, Romania; carmen.pantis@didactic.uoradea.ro (C.P.); brata.roxanadaniela@didactic.uoradea.ro (R.D.B.); 2 Department of Preclinical Disciplines, Faculty of Medicine and Pharmacy, University of Oradea, 1 Decembrie, 410028 Oradea, Romania; alinatirb@uoradea.ro; 3 Department of Internal Medicine II, Diabetes Mellitus, Clinical County Emergency Hospital of Oradea, 410167 Oradea, Romania; 4 Pharmacy Department, Faculty of Medicine and Pharmacy, University of Oradea, 1 University Street, 410087 Oradea, Romania

**Keywords:** organ support, mechanical ventilation, inotropic support, hemodialysis, disease severity, mortality, critical illness, risk stratification, hospital outcomes

## Abstract

**Background**: Assessing disease severity in hospitalized patients is essential for risk stratification and clinical decision-making. While formal severity scores are not always available in routine practice, organ support requirements may serve as pragmatic markers of critical illness. This study evaluated organ support modalities as indicators of disease severity and their association with in-hospital mortality. **Methods**: This retrospective observational study included 1332 adult hospitalized patients managed in either the intensive care unit or medical wards. Data on demographics, clinical complications, organ support requirements (mechanical ventilation, inotropic support, and hemodialysis), and outcomes were extracted from medical records. Cumulative organ support burden was categorized as none, single-organ, or multi-organ support. In-hospital mortality was the primary outcome. **Results**: Mechanical ventilation was required in approximately 71% of patients, inotropic support was required in 31%, and hemodialysis was required in 8%. Mortality was markedly higher among patients requiring organ support compared to those who did not. Mortality reached 70.4% in ventilated patients, 85.1% in those receiving inotropes, and 84.8% in those undergoing hemodialysis. A clear dose–response relationship was observed, with mortality increasing from 3.9% in patients requiring no support to 54.9% in those requiring one modality and 87.6% in those requiring multiple supports. **Conclusions**: Organ support requirements were strongly associated with in-hospital mortality and may serve as pragmatic prognostic indicators in retrospective datasets where formal severity scores are unavailable. However, given that organ support is initiated in response to clinical deterioration, these variables should be interpreted primarily as markers of prognostic association rather than independent severity measures.

## 1. Introduction

Hospitalized patients with acute medical conditions often present with varying degrees of physiological instability and organ dysfunction. Early recognition of disease severity is critical for timely intervention, appropriate triage, and efficient use of healthcare resources. However, accurately assessing severity in real-world clinical settings remains challenging, particularly when standardized scoring systems are not routinely available [1,2,3,4].

Organ support interventions such as mechanical ventilation, vasoactive therapy, and renal replacement therapy are typically initiated in response to life-threatening organ failure. These therapies are, therefore, closely linked to disease severity and are commonly used in critical care practice as indicators of clinical deterioration. Unlike formal severity scores, organ support requirements are simple to identify and consistently documented in medical records, making them potentially valuable for retrospective severity assessment [5,6,7].

Previous studies have demonstrated that individual forms of organ support are associated with poor outcomes, especially in critically ill populations. Nevertheless, many investigations focus primarily on ICU cohorts or rely on complex severity scoring systems. Less attention has been given to evaluating organ support requirements as pragmatic severity markers across broader hospitalized populations that include both ICU and ward patients [8,9,10].

Moreover, the cumulative burden of organ support may provide additional prognostic information. A stepwise increase in the number of failing organs is biologically plausible as a driver of worse outcomes, yet the concept of “organ support burden” as a clinical severity framework remains underexplored in general hospital cohorts [11,12,13].

In many health systems, validated severity scores such as SOFA, qSOFA, or APACHE are not consistently documented outside research settings. Their calculation requires multiple physiological and laboratory variables that may not be simultaneously available, particularly in resource-limited or high-workload environments. In contrast, organ support interventions are routinely recorded, highly standardized, and reflect clinically meaningful deterioration. For this reason, organ support requirements may serve as pragmatic severity surrogates when formal scoring systems are unavailable or incompletely captured.

Therefore, the present study aimed to evaluate organ support requirements as markers of critical illness in hospitalized patients. Specifically, we sought to (1) describe the distribution of major organ support modalities, (2) assess their association with in-hospital mortality, and (3) explore the dose–response relationship between cumulative organ support burden and mortality.

## 2. Materials and Methods

### 2.1. Study Design and Setting

This retrospective observational study was conducted in a tertiary-care hospital. Consecutive adult patients admitted between June 2023 and December 2024 were identified through the hospital electronic admission registry. Consecutiveness was defined as inclusion of all eligible admissions meeting criteria during the study window. Of the included patients, 54.1% were primarily managed in the ICU and 45.9% were managed in medical wards. The study aimed to evaluate organ support requirements as markers of disease severity and their association with clinical outcomes. Missingness was <0.7% for all variables; the highest rates were for diabetes (0.6%) and acute renal failure (0.5%).

Both patients admitted to the intensive care unit (ICU) and those managed in medical wards were eligible for inclusion to reflect real-world clinical practice.

Because the electronic records did not reliably capture exact timing of organ support initiation, support variables reflected whether the intervention occurred at any time during hospitalization.

### 2.2. Study Population

All adult hospitalized patients with available clinical and outcome data were considered eligible. Patients with missing mortality data were excluded. No additional exclusion criteria were applied in order to preserve the representativeness of the cohort.

### 2.3. Data Collection

Clinical and demographic data were extracted from electronic medical records. The following variables were collected:

- Age and sex;
- Comorbidities, including diabetes mellitus;
- Acute complications (e.g., sepsis, pneumonia, septic shock, acute respiratory failure, acute renal failure, metabolic and respiratory acidosis);
- Organ support modalities were as follows:
- Mechanical ventilation refers to invasive mechanical ventilation, whereas non-invasive ventilation was not systematically recorded and could not be reliably analyzed;
- Inotropic/vasoactive support;
- Hemodialysis;
- ICU length of stay;
- In-hospital mortality.

All variables represented routinely documented clinical information.

Acute complications were retrospectively extracted from physician-documented diagnoses.

Organ support interventions were delivered according to institutional standards of care. However, initiation of mechanical ventilation, vasoactive therapy, and renal replacement therapy ultimately remained at the discretion of the treating clinicians. Mechanical ventilation referred to invasive ventilation delivered in the ICU or intermediate care areas. Short-term procedural or operating room ventilation was not counted as organ support.

The ≥1 h threshold was chosen to exclude brief bolus use while retaining clinically meaningful vasoactive exposure. However, we acknowledge that shorter infusions may capture transient hemodynamic instability rather than sustained shock.

Initiation thresholds for mechanical ventilation, vasoactive therapy, and renal replacement therapy followed standard institutional protocols and clinician judgment. Because of the retrospective design, detailed physiological triggers and exact duration of support could not be reliably extracted.

### 2.4. Definitions

Organ support was defined as the use of:

- Mechanical ventilation for respiratory failure.
- Inotropic/vasoactive support was defined as continuous intravenous administration of vasopressors or inotropes (e.g., norepinephrine, epinephrine, dopamine, dobutamine) for ≥1 h for hemodynamic instability (e.g., norepinephrine, epinephrine, dopamine, dobutamine).
- Hemodialysis included both intermittent hemodialysis and continuous renal replacement therapy. Chronic dialysis patients were identified based on documented end-stage kidney disease and maintenance dialysis in prior medical history and were excluded from the hemodialysis exposure group.

Cumulative organ support burden was calculated as the total number of support modalities required per patient. Patients were categorized into three groups:

- No organ support;
- Single-organ support;
- Multi-organ support (≥2 modalities).

Whenever possible, diagnoses of sepsis and septic shock were aligned with Sepsis-3 criteria as documented in the medical record.

### 2.5. Outcomes

The primary outcome was in-hospital mortality, defined as death occurring during hospitalization in either the ICU or ward.

Secondary outcomes included the distribution of organ support modalities and their association with clinical complications.

### 2.6. Statistical Analysis

Continuous variables were expressed as mean ± standard deviation or median as appropriate. Categorical variables were presented as counts and percentages. Analyses were performed using SPSS version 30 (IBM Corp., Armonk, NY, USA).

Group comparisons were performed using Student’s *t*-test or chi-square tests, as appropriate. The dose–response relationship between cumulative organ support burden and mortality was evaluated by stratifying patients according to the number of support modalities.

Normality was assessed using the Shapiro–Wilk test.

Multivariable logistic regression was performed to assess the independent association between organ support modalities and in-hospital mortality, adjusting for age, sex, diabetes, sepsis, septic shock, acute respiratory failure, and ICU admission. Organ support burden was also modeled as an ordinal variable, confirming a significant trend across categories.

A two-sided *p*-value < 0.05 was considered statistically significant. Statistical analyses were performed using standard statistical software. All statistical tests were two-tailed, and statistical significance was defined as *p* < 0.05. Statistical analyses were performed using IBM SPSS Statistics, Version 30 (IBM Corp., Armonk, NY, USA).

Variables with missing data were rare (<0.7%) and were handled by complete-case analysis.

### 2.7. Ethical Considerations

The study was conducted in accordance with the Declaration of Helsinki. Given the retrospective design and use of anonymized data, informed consent was waived. Institutional approval was obtained according to local regulations.

## 3. Results

### 3.1. Baseline Characteristics of the Study Population

A total of 1332 hospitalized patients were included in the analysis. The mean age was 68 years, reflecting a predominantly older adult population. Male patients represented a slight majority of cases.

Sensitivity analyses stratified by ICU versus ward management showed similar directions of association between organ support modalities and mortality, although effect sizes were larger in ICU patients, as expected.

A substantial proportion of patients presented with significant comorbidities, particularly diabetes mellitus. Acute complications at admission or during hospitalization were frequent, including sepsis, pneumonia, and varying degrees of organ dysfunction.

Organ support interventions were commonly required. Mechanical ventilation was the most frequent modality, followed by inotropic support and hemodialysis. These interventions were used across both ICU and ward settings, highlighting the presence of clinically unstable patients outside the ICU environment.

The baseline characteristics of the study population are summarized in Table 1. The cohort consisted predominantly of older adults with a balanced sex distribution. Acute respiratory failure and infectious complications were common. Organ support interventions, particularly mechanical ventilation, were frequently required, reflecting a high burden of severe illness.

### 3.2. Sensitivity Analysis by Care Setting

Sensitivity analyses stratified by ICU versus ward management showed similar directions of association between organ support modalities and mortality. Among ICU patients, mechanical ventilation, inotropic support, and hemodialysis remained strong independent predictors of mortality. The model in ward patients was limited by lower event rates and consequently reduced statistical power; however, effect directions were numerically similar (Table 2).

### 3.3. Organ Support Distribution and Clinical Correlates

Organ support interventions were frequently required in this hospitalized cohort, reflecting a high burden of severe illness. Organ support was recorded if initiated at any time during hospitalization. Timing relative to admission was not consistently available and was not analyzed.

Mechanical ventilation was the most commonly used modality, required in approximately 71% of patients. Inotropic support was administered in about 31% of patients, while 7–8% of patients required hemodialysis. The high proportion of mechanically ventilated patients likely reflects the tertiary-care profile of the hospital and the inclusion of a substantial number of critically ill referrals. This distribution suggests that the cohort represents a high-acuity hospitalized population rather than a general medical cohort.

Patients requiring organ support frequently exhibited markers of severe systemic illness. Sepsis and septic shock were common among patients receiving any form of support. Acute respiratory failure was particularly prevalent, affecting roughly three-quarters of patients across all support modalities.

These findings suggest that organ support requirements may serve as practical indicators of clinical severity (Table 3).

**Figure 1:**
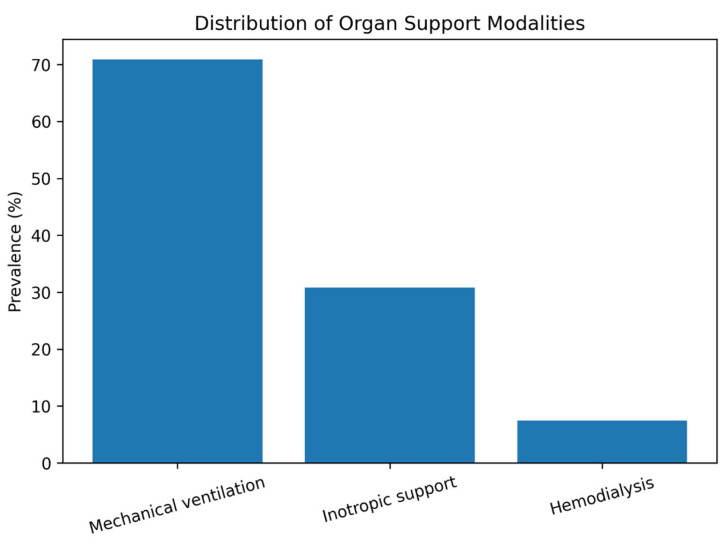
Distribution of organ support modalities among hospitalized patients.

The distribution of organ support modalities is shown in Figure 1, showing the predominance of mechanical ventilation in this cohort.

### 3.4. Organ Support and Mortality Risk

The requirement for organ support was strongly associated with in-hospital mortality.

Patients requiring mechanical ventilation had markedly higher mortality compared to those not requiring ventilation (70.4% vs. 5.4%). Similarly, mortality was substantially higher among patients receiving inotropic support (85.1% vs. 36.6%) and hemodialysis (84.8% vs. 48.8%).

These findings demonstrate a clear association between advanced life-support interventions and poor outcomes, reflecting the severity of underlying organ dysfunction. The gradient was particularly pronounced for circulatory and renal support modalities, which were associated with the highest mortality rates (Table 4).

Overall, organ support requirements emerged as strong clinical markers of adverse prognosis.

**Figure 2:**
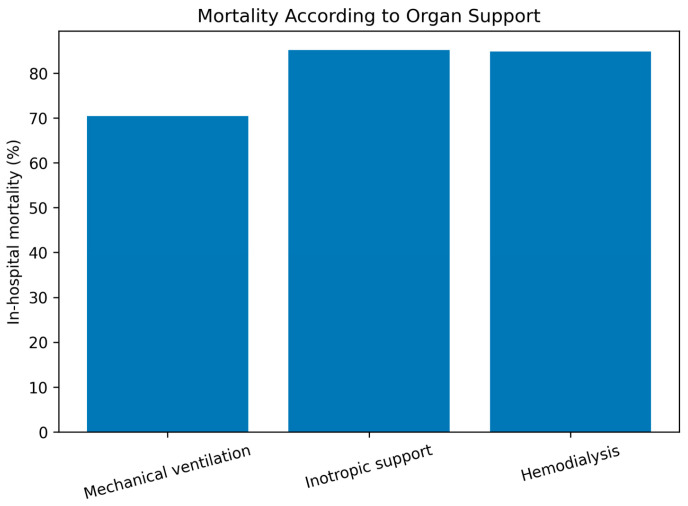
In-hospital mortality according to major organ support modalities. Bars represent the proportion (%) of patients who died during hospitalization among those who required mechanical ventilation, inotropic/vasoactive support, or hemodialysis. Mortality was highest in patients requiring circulatory and renal support.

As shown in Figure 2, mortality was markedly higher among patients requiring any form of organ support, particularly inotropic support and hemodialysis.

### 3.5. Dose–Response Effect of Cumulative Organ Support

A stepwise association consistent with a dose–response pattern was observed between the number of organ support modalities and in-hospital mortality.

Patients requiring no organ support had very low mortality (3.9%). Mortality increased sharply among those requiring one organ support modality (54.9%) and reached extremely high levels in patients requiring two or more supports (87.6%).

This stepwise gradient indicates that cumulative organ support burden strongly reflects disease severity and prognosis (Table 5).

**Figure 3:**
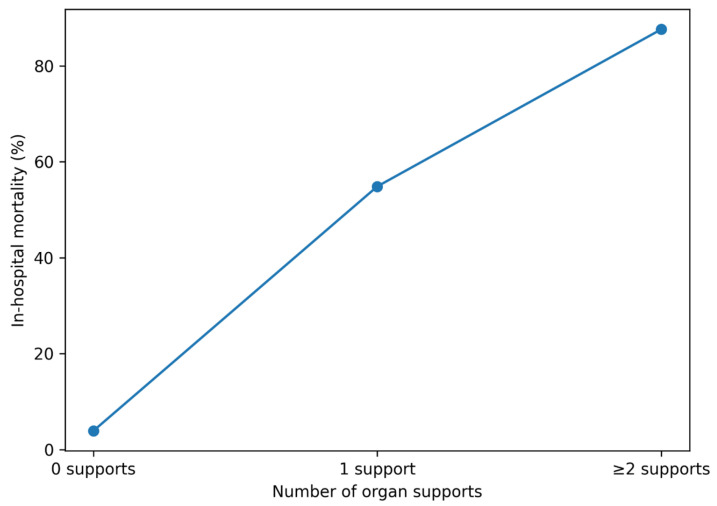
Dose–response relationship between cumulative organ support burden and in-hospital mortality. Mortality increased stepwise with the number of required organ support modalities.

As illustrated in Figure 3, a marked dose–response relationship was observed, with mortality increasing progressively according to cumulative organ support burden.

### 3.6. Multivariable Analysis

In multivariable logistic regression adjusting for age, sex, diabetes, and ICU admission, organ support modalities remained consistently associated with mortality.

Mechanical ventilation was the strongest independent predictor (aOR 31.95, 95% CI 19.47–52.43, *p* < 0.001). Inotropic support (aOR 4.98, 95% CI 3.52–7.03, *p* < 0.001) and hemodialysis (aOR 5.55, 95% CI 2.57–11.99, *p* < 0.001) also showed robust independent associations. After adjustment, mechanical ventilation, inotropic support, and hemodialysis remained independently associated with mortality.

Older age and diabetes were modest but significant predictors, while sex and ICU admission were not independently associated after adjustment (Table 6).

## 4. Discussion

This study demonstrates that organ support requirements are strong and clinically meaningful markers of disease severity among hospitalized patients. The need for mechanical ventilation, inotropic support, and hemodialysis was consistently associated with markedly higher mortality, highlighting the prognostic value of routinely recorded life-support interventions. These findings suggest that the prognostic relevance of organ support requirements is present across different care environments, not exclusively in ICU populations.

### 4.1. Organ Support as a Reflection of Disease Severity

Organ support therapies are typically initiated in response to life-threatening organ dysfunction. In our cohort, patients requiring any form of support exhibited substantially higher mortality compared to those managed without advanced support. Cumulative organ support burden may help clinicians rapidly recognize patients at high risk of deterioration and anticipate resource needs. This finding reinforces the concept that organ support requirements may act as pragmatic clinical surrogates in real-world settings [14,15]. Because timing was not assessed, supports may represent both baseline severity and in-hospital deterioration.

Intervention thresholds may differ between ICU and ward settings, influenced by monitoring intensity, staffing, and resource availability. Such variability may partly explain differences in outcomes and should be considered when interpreting associations.

Mechanical ventilation was the most frequently required modality and was associated with a pronounced increase in mortality. This likely reflects the severity of respiratory failure and systemic disease processes, including sepsis and metabolic derangements. Similarly, the need for vasoactive support indicates circulatory instability and impaired perfusion, both well-recognized predictors of poor outcomes [16,17,18,19,20,21]. The relatively high prevalence of mechanical ventilation likely reflects the tertiary-care and referral profile of our institution, which manages a large proportion of critically ill patients. Therefore, the cohort should be interpreted as a high-acuity hospitalized population rather than a general medical cohort.

The high prevalence of mechanical ventilation partly reflects the tertiary referral profile and inclusion of critically ill transfers. Some ventilated patients were managed outside the ICU in intermediate-care areas, which may explain the discrepancy between ICU admission rates and ventilation prevalence.

The consistency of findings in ICU-stratified analyses supports the robustness of the observed associations across care settings, although statistical power was lower in ward-only models.

### 4.2. Circulatory and Renal Support as High-Risk Indicators

The highest mortality rates were observed among patients receiving inotropic support and hemodialysis. These modalities often signal advanced multi-organ dysfunction and hemodynamic compromise. Renal replacement therapy, in particular, is commonly associated with severe systemic illness and inflammatory states [22,23,24,25].

Our findings align with the prior critical care literature, indicating that renal and circulatory failure carry particularly poor prognostic implications. Importantly, these interventions are easily identifiable in clinical records, making them practical markers for bedside risk stratification.

### 4.3. Dose–Response Relationship and Cumulative Burden

A key contribution of this study is the demonstration of a clear stepwise association between cumulative organ support burden and mortality. Mortality increased stepwise from patients requiring no support to those requiring single and multiple support modalities [26,27,28].

An important consideration is that disease severity at admission and pre-existing comorbidities may influence both the need for organ support and mortality risk. Although we adjusted for major factors such as age and diabetes, residual confounding is likely. Organ support should therefore be interpreted as a marker of clinical trajectory rather than a causal determinant of outcome.

This gradient supports the biological plausibility of cumulative organ dysfunction driving adverse outcomes. The concept of “organ support burden” may, therefore, provide a simple framework for severity classification when formal scoring systems are unavailable [29].

Importantly, the association between organ support and mortality persisted after multivariable adjustment, supporting their role as independent prognostic indicators rather than merely reflections of baseline differences.

Such an approach could be particularly useful in resource-limited environments or retrospective analyses where standardized severity scores are not routinely collected.

### 4.4. Clinical Implications

From a clinical perspective, early identification of patients likely to require organ support could facilitate proactive management and escalation of care. Organ support burden may also help guide resource allocation and prioritization in high-demand settings [30,31,32]. Cumulative organ support burden could be operationalized as a simple bedside stratification tool in retrospective audits or quality-improvement initiatives. For example, tracking escalation from single- to multi-organ support may help flag patients at high risk of deterioration and guide timely escalation of care.

Moreover, these findings underscore the importance of dynamic patient monitoring, as progression from single-organ to multi-organ support appears to correspond with sharply rising mortality risk.

### 4.5. Strengths and Limitations

This study benefits from a large real-world cohort reflecting routine clinical practice. The use of readily available clinical variables enhances applicability and reproducibility.

A major limitation is confounding by indication: organ support is not only a marker but also a consequence of severity. Thus, the observed associations may partly reflect clinical decision-making triggered by worsening illness. This limits causal interpretation, and there is a lack of granular physiological data, such as vital signs or laboratory-based severity scores.

A key limitation is the lack of reliable temporal data regarding initiation of organ support relative to hospital admission. Therefore, organ support burden likely reflects a combination of baseline severity at presentation and in-hospital clinical deterioration. This introduces the possibility of reverse causation and limits interpretation of organ support as an early prognostic marker.

However, several limitations should be acknowledged. The retrospective design precludes causal inference and may introduce residual confounding. Formal severity scores such as SOFA or APACHE were not available for comparison. Additionally, single-center data may limit generalizability. Our intention is not to replace validated severity scores such as SOFA or APACHE, but to propose a pragmatic surrogate when such scores are unavailable.

### 4.6. Future Directions

Prospective studies incorporating standardized severity scoring systems and longitudinal assessment of organ support trajectories are warranted. Evaluating temporal patterns of organ support escalation may further refine prognostic models.

Taken together, our findings suggest that organ support requirements represent robust and clinically accessible markers of disease severity. The cumulative burden of organ support provides a risk stratification aid that is closely linked to mortality risk.

Future studies should evaluate the temporal dynamics of organ support initiation, distinguishing early versus late escalation. Such analyses may help differentiate baseline severity from deterioration trajectories and refine prognostic models.

## 5. Conclusions

Organ support requirements are strongly associated with disease severity and in-hospital mortality among hospitalized patients. Mechanical ventilation, vasoactive support, and hemodialysis were each associated with markedly increased in-hospital mortality, underscoring their prognostic relevance.

A clear dose–response relationship was observed, with mortality rising steeply as the number of required organ support modalities increased. This cumulative organ support burden provides a simple and clinically applicable framework for severity stratification when formal scoring systems are unavailable.

Recognizing organ support requirements as indicators of severity may improve risk assessment, guide clinical decision-making, and support more efficient resource allocation in acute care settings. Future prospective studies should validate these findings and explore how dynamic changes in organ support needs influence outcomes.

## Figures and Tables

**Table 1:** Baseline Characteristics Stratified by In-Hospital Mortality.

Variable	Survivors	Non-Survivors	*p*-Value
Age, mean ± SD (years)	65.5 ± 13.2	70.3 ± 12.5	<0.001
Male sex, %	50.9	49.9	0.736
Diabetes mellitus, %	28.3	36.3	0.002
Acute complications			
Sepsis, %	12.4	22.2	<0.001
Pneumonia, %	21.1	29.9	<0.001
Septic shock, %	3.6	14.4	<0.001
Acute respiratory failure, %	51.4	76.7	<0.001
Acute renal failure, %	15.9	35.4	<0.001
Organ support			
Mechanical ventilation, %	43.2	96.9	<0.001
Inotropic support, %	9.4	50.9	<0.001
Hemodialysis, %	2.3	12.2	<0.001

Continuous variables are presented as mean ± standard deviation (SD) and categorical variables as percentages (%). *p*-values were calculated using Student’s *t*-test for continuous variables and chi-square tests for categorical variables. SD, standard deviation.

**Table 2:** Sensitivity analysis: multivariable logistic regression of factors associated with in-hospital mortality among ICU patients.

Variable	aOR	95% CI	*p*
Age	1.03	1.01–1.04	<0.001
Septic shock	4.25	1.87–9.65	<0.001
Mechanical ventilation	21.96	11.24–42.91	<0.001
Inotropic support	3.40	2.20–5.26	<0.001
Hemodialysis	4.34	1.63–11.52	0.003

aOR = adjusted odds ratio; CI = confidence interval; ICU = intensive care unit. The model was restricted to ICU patients and included age, septic shock, mechanical ventilation, inotropic support, and hemodialysis as covariates entered simultaneously. Odds ratios represent independent associations with in-hospital mortality. A two-sided *p*-value < 0.05 was considered statistically significant.

**Table 3:** Clinical Correlates of Organ Support Modalities.

Organ Support	Sepsis (%)	Acute Respiratory Failure (%)	Septic Shock (%)
Mechanical ventilation	19.6	76.6	11.7
Inotropic support	23.7	75.1	15.1
Hemodialysis	26.3	76.8	15.2

Values are presented as percentages (%) representing the prevalence of each complication among patients receiving the specified organ support modality.

**Table 4:** In-Hospital Mortality According to Organ Support.

Organ Support	Mortality with Support (%)	Mortality Without Support (%)
Mechanical ventilation	70.4	5.4
Inotropic support	85.1	36.6
Hemodialysis	84.8	48.8

Values are presented as percentages (%). Mortality rates represent the proportion of in-hospital deaths among patients with and without the specified organ support modality.

**Table 5:** Mortality According to Cumulative Organ Support.

Number of Organ Supports	*n*	Mortality (%)
0 supports	357	3.9
1 support	556	54.9
≥2 supports	419	87.6

Values are presented as counts (*n*) and percentages (%). Mortality refers to in-hospital mortality within each category of cumulative organ support.

**Table 6:** Multivariable logistic regression analysis of factors associated with in-hospital mortality.

Variable	aOR	95% CI	*p*
Age	1.023	1.011–1.034	<0.001
Male	0.78	0.58–1.05	0.10
Diabetes	1.44	1.04–1.99	0.027
Sepsis	0.88	0.55–1.41	0.59
Septic shock	3.10	1.57–6.15	0.001
Acute respiratory failure	1.22	0.87–1.71	0.24
Acute renal failure	1.43	0.99–2.07	0.054
ICU admission	0.99	0.73–1.33	0.93
Mechanical ventilation	28.69	17.23–47.77	<0.001
Inotropic support	4.74	3.34–6.71	<0.001
Hemodialysis	4.62	2.06–10.34	<0.001

aOR = adjusted odds ratio; CI = confidence interval; ICU = intensive care unit. All variables were entered simultaneously into the model. The model was adjusted for age, sex, diabetes mellitus, sepsis, septic shock, acute respiratory failure, acute renal failure, ICU admission, mechanical ventilation, inotropic support, and hemodialysis. Odds ratios represent independent associations with in-hospital mortality. A two-sided *p*-value < 0.05 was considered statistically significant.

## Data Availability

The data presented in this study are available on request from the corresponding author. The data are not publicly available due to privacy or ethical restrictions.

## Abbreviations

The following abbreviations are used in this manuscript:

ICU	Intensive Care Unit
LOS	Length of Stay
OR	Odds Ratio
CI	Confidence Interval
SD	Standard Deviation
RRT	Renal Replacement Therapy
